# Seasonal Fluctuations of Microbial Aerosol in Live Poultry Markets and the Detection of Endotoxin

**DOI:** 10.3389/fmicb.2017.00551

**Published:** 2017-03-30

**Authors:** Bo Wu, Kai Meng, Liangmeng Wei, Yumei Cai, Tongjie Chai

**Affiliations:** ^1^College of Veterinary Medicine, Shandong Agricultural University, Sino-German Cooperative Research Centre for Zoonosis of Animal Origin Shandong ProvinceTai’an, China; ^2^Collaborative Innovation Centre for the Origin and Control of Emerging Infectious Diseases of Taishan Medical CollegeTai’an, China

**Keywords:** live poultry market, cultural airborne bacteria, airborne fungi, Gram-negative bacteria, airborne endotoxin, public health significance

## Abstract

Microbial aerosol whose species and concentrations are closely related to human health is ubiquitous. The effect of microbes on human and animal health and production performance is, in many cases, caused by the spreading of air. Samples in this experiment were from a live poultry market (LPM) in Tai’an, China, collected three times a day (8 am, 14 pm, and 20 pm) over three consecutive days each month for 11 months (Original sampling plan was a year, the government due to the environmental protection, the was LPM closed). The main indicators of the test were concentrations of cultural airborne bacteria, airborne fungi, and Gram-negative bacteria. At the same time the species of Gram-negative bacteria and the concentration of endotoxin were tested. Temperature and humidity were recorded in the process of each sampling. The results showed that the diurnal variation of the concentration of bacteria, fungi, and Gram-negative bacteria is higher in the morning and evening, but lower at noon. The concentrations of airborne bacteria and Gram-negative bacteria increased in earlier months and decreased in later months, with the peak appearing in the autumn. The concentration of fungi showed a decrease first and then tended to stabilize, with the peak occurring in the spring. The concentration peak of endotoxin occurred in the summer. Endotoxin levels were significantly correlated with humidity (*r* = 0.90, *p* < 0.01). Most bacteria were distributed at the third and fourth stages (2.1–4.7 μm) in the ANDERSEN-6 sampler. The dominant species of Gram-negative bacteria during the four seasons were *Pseudomonas aeruginosa, Acinetobacter, Klebsiella pneumoniae*, and *Salmonella*. In China, people have a habit of eating fresh poultry, LPM distribution is widespread, stream of people and traffic flow are large, easily caused the spread of bacteria and viruses, so the LPM microbial aerosol research have significant public health implications.

## Introduction

Ambient air is filled with microbial aerosols, which mainly include bacteria, fungi, viruses, and pollen grains. Airborne microbes are considered as one of the most important contaminants, which get more and more attention (Kawther, 2002). Microbial aerosols can spread a variety of diseases, and are likely to cause a variety of respiratory diseases and allergic reactions in particular (Zhao, 2001; Haas et al., 2013). Microbial aerosols pose a potential threat to the health of animals and humans by invading the body through mucosa, skin damage, the digestive tract, and the respiratory tract (Duan, 2005; Wen et al., 2013). The distribution of airborne bacterial aerosol in live poultry market (LPM), on the one hand, is affected by the sale of live poultry and the flow of vendors and customers; on the other hand, it is influenced by natural factors such as traffic and meteorological events (Liang et al., 2013). A number of studies on the distribution of bacteria indoors and outdoors have been investigated. Now the focus has gradually transferred to the public environment, like an investigation on the distribution of bacteria and related environmental factors in subway stations (Hwang et al., 2010), airborne bacteria and fungi in factories (Kim et al., 2009), and airborne bacteria in outdoor environments of cities (Shaffer and Lighthart, 1997). Distribution of bacteria on the 5–6 stage of the ANDERSEN-6 sampler (equivalent to PM2.5) is about 20%, they can enter the respiratory tract and alveolar, easily lead to respiratory infections, it is very harmful to people.

Airborne fungi, included in the PM2.5 range, distribute extensively as an important part of the urban ecosystem, in a great amount and with a large number of species (Li and Kuo, 1994). Airborne fungi can cause a variety of fungal diseases and toxin poisoning in livestock and poultry. Its widespread existence has posed a great threat to the livestock and poultry breeding industry (Fukutomi, 2016). Therefore, the control of fungal aerosol and its toxin levels in the culture environment can effectively reduce the harm to the cultured animals. Airborne fungal spore species are very rich, in which more than 40,000 species are known to exist, but only a small part can be cultured on a medium (O’Connor et al., 2004). The dominant genera of airborne fungi are *Cladosporium, Penicillium, Aspergillus*, and *Alternaria* etc. Among them, *Cladosporium* is most abundant and can reach 75% of the total number of airborne fungi, but concentrations differ in different climatic conditions and ecological environments (Airaudi and Marchisio, 1996). Fungal Aerosols in LPM extend and spread through the air. High concentrations of fungal aerosols cause environmental biological contamination in the environment, it threatens the health of human and animals.

Gram-negative bacteria only account for a small proportion of bacterial aerosol, but a large number of pathogenic bacteria and conditional pathogenic bacteria are contained in this (Stojek et al., 2008). Thus, the concentration and composition is of great significance for public environmental quality. Bacterial endotoxin is from lipopolysaccharide protein complex on the outer membrane of Gram-negative bacteria. It has an extensive impact on human and animal body cells and humoral immune response (Park et al., 2001). It can lead to dramatic changes in the number of white blood cells in the body, which leads to disorders in immune function (Shang et al., 2016). Some studies indicate that airborne endotoxin is an important component of airborne biological aerosol, and can produce many toxic effects on the body, such as heat source, leukopenia, Shwartzman reaction, endotoxemia, shock, and so on (Solov’eva and Ovodov Iu, 1983). Dutkiewicz (1994) also point out that workers with long-term exposure to toxins in the environment of high animal husbandry are potentially in danger of being attacked by respiratory tract disease. In addition, according to many scholars reported that endotoxins may affect human fluid and cellular immunities, and potentially influence the lungs. All of the above indicate the necessity and importance of airborne endotoxin investigation. In order to assess the risk to human and animal, the concentration of Gram-negative bacteria and endotoxin was tested in LPM.

In China, people have a habit of cooking poultry soon after it has been slaughtered, so LPM is relatively common and have a large number of customers. Research on microbial aerosol in LPM can fully understand the regional status of microbial aerosol. This is beneficial for controlling microbial aerosol pollution, improving the regional environment, and protecting human health. The LPM microbial aerosol research have significant public health implications.

## Materials and Methods

### Selection of Sampling Sites

The sampling location is in a downtown LPM in Tai’an City, which demonstrates the typical characteristics of a monsoon climate, with two climatic seasons: a dry season from November to April and a rainy season from May to October. There is a great deal of live poultry slaughter and trade every day. According to observations, a large amount of live poultry is transported to the market at night and is slaughtered and sold the next day. Owing to the frequent slaughtering, trade, and the huge flow of people, the air quality is relatively poor.

### Sample Collection

#### Collection of Cultural Airborne Bacteria, Airborne Fungi, and Gram-negative Bacteria

Cultural airborne bacteria, airborne fungi, and Gram-negative bacteria were collected using an international standard ANDERSEN-6 sampler. Each stage of the sampler included a plate with 400 holes of uniform diameter, through which air was drawn at a speed of 28.3 l min^-1^, with 5% male sheep blood agar culture base, a sand fort weak training base, and a Gram-negative bacteria selective culture base as a sampling medium. The collector was set in the center of the LPM, 80–100 cm high in the air. Driving times were different according to different months, being 1–2, 3–5, and 15–25 min, respectively. The number of colonies per class was approximately 30–300. Airborne particles were divided into six parts, whose aerodynamic diameters were 7.0 μm (stage 1), 4.7–7.0 μm (stage 2), 3.3–4.7 μm (stage 3), 2.1–3.3 μm (stage 4), 1.1–2.1 μm (stage 5), and 0.65–1.1 μm (stage 6).

#### Collection of Endotoxin

The international standard ANDERSEN-6 level impact type air microorganism sample collector was used to collect endotoxin. The air flow rate was 12.5 L/min, 50 mL water without a heat source was used as the sampling medium, and the drive time was 15 min.

### Sampling Time

Sample collection was performed in three randomly chosen consecutive days each month for 11 months (from June 2014 to April 2015). In each day, samples were collected at 8 am, 14 pm, and 20 pm, respectively.

### Measurement of Environmental Factors

The temperature and humidity of each sample were recorded by hygrothermograph.

### Culture, Counting, and Identification of Samples

The colony count was collected after the cultural airborne bacteria plate was cultured in a 37°C incubator for 24–48 h, and the content of cultural airborne bacteria (CFU/m^3^) was calculated according to Andersen’s calibration.

The airborne fungi collection plate was placed and cultured in an incubator at 25°C for 72 h, and then the number of fungal colonies on the sample dish at each stage was calculated. An adjusted counting was conducted after they were cultured 7 days in order to avoid errors resulting from delayed colonies. The result obtained was the number of fungal aerosol particles.

Gram-negative bacteria selective medium plates were collected from incubators for a colony count 24–48 h later, on which the colonies were gram stained to distinguish Gram-negative and positive bacteria, according to Andersen’s correction table for the correction calculation of Gram-negative bacteria content (CFU/m^3^). Then one of the Gram-negative bacteria were identified using a biochemical identification system (Gram-negative bacteria identification system, Qingdao haibo Biological).

The formula for calculating units of colonies in per cubic meter of air is: CFU/m^3^ = [Andersen-6 stage sampler to collect the colony total/28.3L/m^3^ × sampling time (min)] × 1 000.

Endotoxin was determined by LAL reagent, and the content of endotoxin in the air was calculated:

Y = (volume concentration of endotoxin in the sample × media volume)/(AGI velocity of air flow × sampling time).

### Statistical Analysis

The median, maximum, and minimum value were computed using Microsoft Excel. Spearman correlation analyses were also used to assess the relationship between Gram-negative bacteria, endotoxin and other environmental factors. All statistical analyses were carried out using the SPSS software package (version 19.0; SPSS Inc., USA).

## Results

### Total Concentration

Samples were collected three times a day for three consecutive days in each month from June 2014 to April 2015. Results indicated that the concentration of bacteria in the atmosphere varies greatly among different sampling months and seasons. Throughout the year, the concentration ranges of bacteria, fungi, and Gram-negative bacteria were 1.84 × 10^3^–5.98 × 10^4^ CFU/m^3^, 6.57 × 10^2^–1.33 × 10^4^ CFU/m^3^, and 51–3.13 × 10^3^ CFU/m^3^, respectively; intermediate values were 1.27 × 10^4^ CFU/m^3^, 3.578 × 10^3^ CFU/m3, and 5.76 × 10^2^ CFU/m3, respectively. The maximum, minimum, and intermediate values for each month, as well as for each quarter, were also listed (see **Table 1**).

**Table 1:** The concentrations of bacteria, fungi, and Gram-negative bacteria.

Strain month	Bacteria (×10^3^ CFU/m^3^)				Fungi (×10^3^ CFU/m^3^)				G^-^ bacteria (×10^2^ CFU/m^3^)			
	Tot	Max	Min	Med	Tot	Max	Min	Med	Tot	Max	Min	Med
Tot	108	59.8	1.8	12.7	108	13.3	0.7	3.6	108	31.3	0.5	5.8
Jan	9	9.4	2.3	4.8	9	13.3	4.6	6.8	9	1.7	0.5	1.4
Feb	9	6.7	4.2	4.6	9	9.1	4.9	7.0	9	2.2	1.6	1.8
Mar	9	8.0	1.8	5.9	9	6.1	1.4	2.4	9	2.5	0.7	1.8
Apr	9	24.1	3.5	8.3	9	7.6	2.2	4.2	9	21.2	1.7	7.2
Jun	9	41.4	28.3	32.6	9	5.3	3.3	4.2	9	11.1	8.1	9.8
Jul	9	59.8	31.4	41.2	9	3.7	2.6	3.0	9	31.3	5.1	6.7
Aug	9	49.7	12.4	33.3	9	4.6	1.7	2.4	9	22.1	4.6	15.2
Sep	9	38.8	12.1	23.6	9	6.6	2.9	3.6	9	18.6	1.5	5.7
Oct	9	48.7	9.3	26.3	9	8.5	1.0	3.6	9	8.3	1.3	5.1
Nov	9	20.1	13.6	17.2	9	3.7	1.8	2.5	9	6.9	4.8	5.3
Dec	9	15.9	5.1	9.3	9	2.1	0.7	1.3	9	8.9	1.1	2.4

*Tot, total samples; Max, maximum; Min, minimum; Med, median.*

### Seasonal Concentration Variations

**FIGURE 1:**
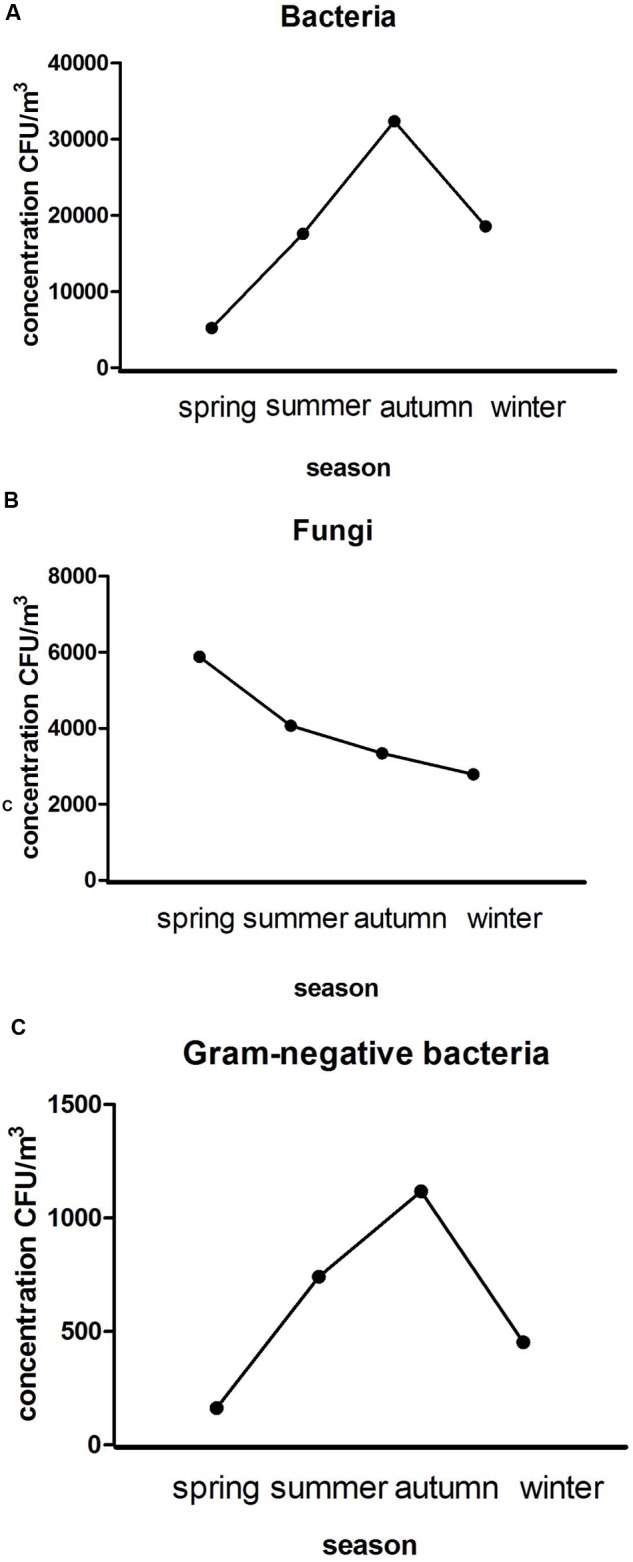
**Average seasonal concentration variations of bacteria (A)**, fungi **(B)**, and Gram-negative bacteria **(C)**.

The average concentrations of aerobic bacteria in the four seasons was significantly different. The peak value, appearing in autumn, was significantly higher than that in the other three seasons (*P* < 0.01), with the average concentration being 3.23 × 10^4^CFU/m^3^ (see **Figure 1A**). The average concentrations of fungi in the seasons were not significantly different. The peak in spring was higher than that in other seasons (*P* > 0.05), with the average concentration being 5.88 × 10^3^ CFU/m^3^ (see **Figure 1B**). The average concentration of Gram-negative bacteria was significantly different. The peak in autumn was significantly higher than that in other seasons (*P* < 0.01), with the average concentration being 1.12 × 10^3^ CFU/m^3^ (see **Figure 1C**).

### Monthly Concentration Variations

**FIGURE 2:**
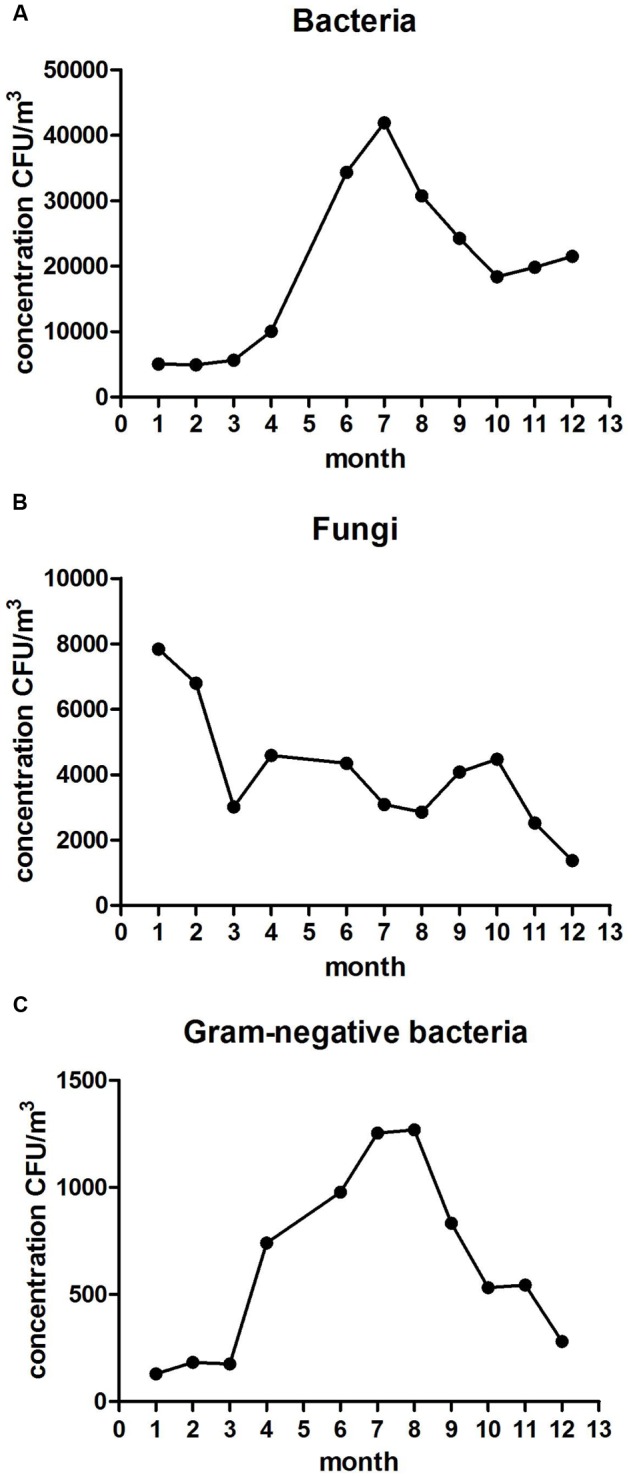
**Average monthly concentration variations of bacteria (A)**, fungi **(B)**, and Gram-negative bacteria **(C)**.

Annual monitoring showed that concentrations of aerobic bacteria from June to September were significantly higher than that in other months (*P* < 0.01), with the highest concentration of 4.20 × 10^4^ CFU/m^3^ in July and the lowest of 4.93 × 10^3^ CFU/m^3^ in February (see **Figure 2A**). The concentrations of fungi from January to February were significantly higher than that in other months (*P* < 0.05). The highest point of 7.84 × 10^3^ CFU/m^3^ was in January and the lowest of 1.37 × 10^3^ CFU/m^3^ in December (see **Figure 2B**). Concentrations of Gram-negative bacteria from June to July were significantly higher than that of other months, with the highest and lowest points in August and January, respectively (see **Figure 2C**).

### Daily Concentration Variations

**FIGURE 3:**
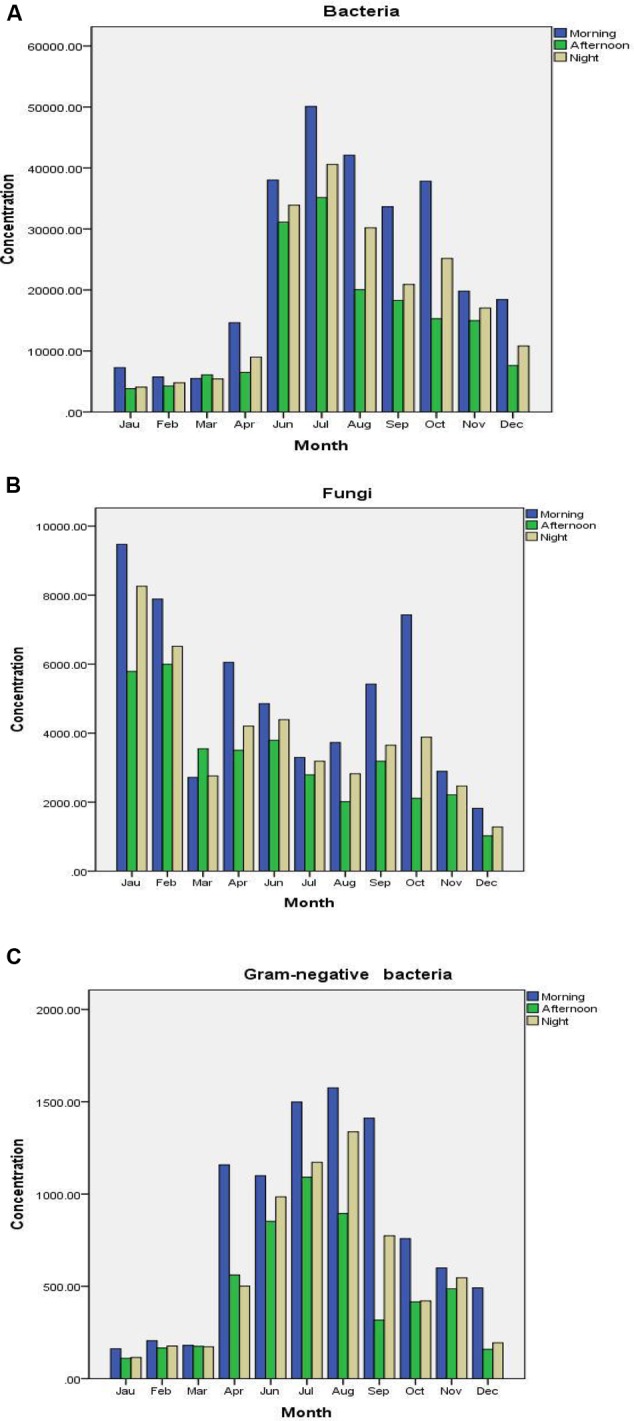
**Daily concentration variations of bacteria (A)**, fungi **(B)**, and Gram-negative bacteria **(C)**.

Aerobic bacteria, fungi, and Gram-negative bacteria were detected three times at 8 am, 14 pm, and 20 pm each day. There was a trend that low peak values generally presented at noon (see **Figure 3**).

### The Hierarchical Distribution Map

**FIGURE 4:**
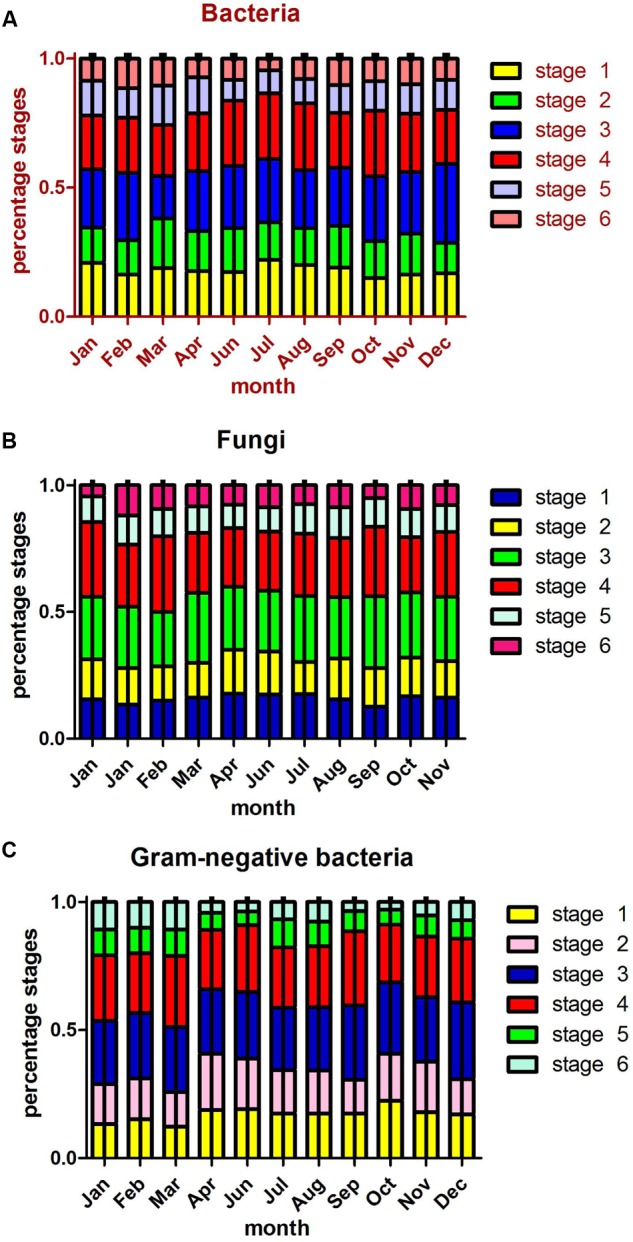
**The Average monthly hierarchical distribution maps of bacteria (A)**, fungi **(B)**, and Gram-negative bacteria **(C)**.

The morphology of bacteria, fungi, and Gram-negative bacteria showed normal distribution. Most of the bacteria were distributed at the third level (3.3–4.7 μm) (*P* < 0.05), and least at the sixth level (0.65–1.1 μm) (*P* < 0.01). The highest distribution rates at level three were 28.8, 29.7, and 30.0%, respectively; the lowest rates at level 6 were 7.2, 4.3, and 3.0%, respectively. Their distributions at levels 3 and 4 were approximately 50%. Other distributions are shown in **Figure 4**.

### Identification of Endotoxin and Gram-negative Bacteria

**FIGURE 5:**
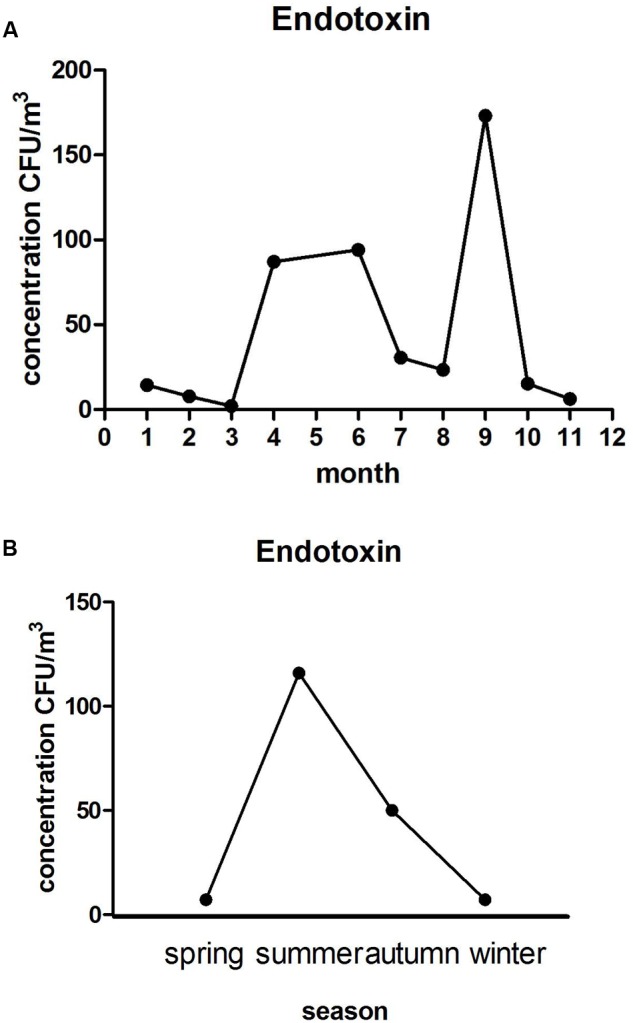
**The monthly (A)** and seasonal **(B)** average concentrations trend of endotoxin.

The concentration of airborne endotoxin in each month of the year is shown in **Figure 5A**, with peak values appearing in September that were significantly different from that in other months (*P* < 0.01). The monthly average was around 2.3–2.03 × 10^2^ EU/m^3^. **Figure 5B** shows concentrations of airborne endotoxin in each season of the year, with the peak of 1.16 × 10^2^ EU/m^3^ in the summer which is significantly different from that in other seasons (*P* < 0.01).

Statistical analysis was carried out on the results of Gram-negative bacteria of the fifth and sixth levels, indicating that dominant bacteria were *Pseudomonas aeruginosa, Acinetobacter, Klebsiella pneumonia*, and *Salmonella*, accounting for 18, 15, 14, and 17%, respectively. A total of 22 Gram-negative bacteria were detected (see **Table 2**).

**Table 2:** Results of Gram-negative bacteria identification.

Spring		Summer		Autumn		Winter	
Genera/species	Per (%)	Genera/Species	Per (%)	Genera/Species	Per (%)	Genera/Species	Per (%)
*P. aeruginosa*	18	*Acinetobacter*	15	*K. pneumoniae*	14	*Salmonella*	17
*K. pneumoniae*	15	*K. pneumoniae*	15	*Salmonella*	12	*Citrobacter Werkman and Gillen*	15
*C. freundii*	13	*Salmonella*	13	*Proteus*	11	*E. agglomerans*	14
*M. Vibrio*	12	*P. aeruginosa*	12	*P. aeruginosa*	11	*P. aeruginosa*	13
*C. luteola*	11	*Citrobacter Werkman and Gillen*	10	*M. Vibrio*	10	*E. coli*	12
*Acinetobacter*	9	*E. coli*	9	*S. Desertification*	8	*V. metschnikovii*	8
*P. fluorescens*	7	*P. putida*	8	*E. coli*	7	*Proteus*	6
*P. putida*	3	*K. oxytoca*	7	*C. luteola*	6	*K. pneumoniae*	5
*Y. enterocolitica*	3	*Proteus*	4	*S. marcescens*	5	*Acinetobacter*	4
*K. ornithinolytica–*	2	*S. Desertification*	2	*S. liquefaciens*	5	Others	6
*S. ficaria*	1	*Others*	5	*Acinetobacter*	4	-	-
others	6	-	-	Others	7	-	-

*Per, percentage.*

### The Relationship between Gram-negative Bacteria, Endotoxin, and Environmental Factors

In this experiment the temperature and humidity of each time point were recorded (see **Table 3**). A weak correlation was found between the relative humidity and the concentration of the Gram-negative bacteria (*P* < 0.05, *r* = 0.3), and a significant correlation was found between the concentration of the endotoxin and that of relative humidity (*P* < 0.01, *r* = 0.9). There was weak correlation between the concentration of airborne endotoxin and that of airborne Gram-negative bacteria (*P* < 0.05, *r* = 0.3).

**Table 3:** Record of temperature and humility.

Mon	1	2	3	4	6	7	8	9	10	11	12
Tem	8.9	5.3	17.3	25.1	32.1	30.6	28.4	28.8	23.1	12.3	8.7
Hum	42.2	39.3	27.7	56.8	66.5	50.1	47.8	73.1	38.4	32.4	30.7

*Mon, month; Tem, temperature; Hum, humility.*

## Discussion

We conducted a comprehensive and systematic monitoring of a LPM for 1 year, which helped us understand the regularity of variations of microbial aerosol in the area. In the past, there have been many studies on the distribution of bacteria and fungi in different time and spaces (Hu et al., 2015), but the study of Gram-negative bacteria and endotoxin is rare. This experiment was conducted to study the bacteria, fungi, Gram-negative bacteria, and endotoxin in a LPM for a whole year. The results showed that the distribution of the bacteria in a LPM was very different during each period of time, and usually with high concentrations.

Previous studies have found that a variety of meteorological factors (wind, temperature, humidity, etc.) have a greater impact on the concentration of bacteria (Fang et al., 2005). The concentration of bacteria in the atmosphere, in addition to being influenced by its own environmental factors, is closely related to human activities, such as the flow of people, flow of traffic, etc (Liang et al., 2013). These human activities can result in the flow of dust and small particles in the soil, causing an increase in the concentration of microbial aerosol (Hameed et al., 2009). Changes of airborne bacteria concentration in the research are mainly related to the microenvironmental conditions, sampling time, climatic conditions, and human activities (Fang et al., 2007). Previously, relative studies have been conducted in many parts of the world. The studies found that the airborne bacteria concentration is higher in the summer mainly due to high temperature (Hameed et al., 2009). Traffic flows in LPM have great growth in summer, and it is easier for bacteria to breed and spread along the ground dust and soil particle diffusion into the atmosphere, causing an increase of microbial aerosol concentration. At three sampling time points, bacterial concentrations at 14 pm were significantly lower than that at 8 am and 20 pm. The main factors affecting the concentration of airborne bacteria during a whole day are temperature, human activity, sunrise, atmospheric stability, wind speed, humidity, and so on (Hwang et al., 2010). At the first sampling time point (8 am), the sun has risen, the temperature begins to rise, radiation enhances, and LPM are open for a day of slaughtering and business. With the increase of customers and vehicles, airborne bacterial aerosol particles are suspended in soil and dust, resulting in a higher concentration of bacteria. At the second sampling time period (14 pm), the local people generally have a lunch break, so the number of vehicles and customers is greatly reduced. In an environment with higher solar radiation and lower relative humidity, airborne bacteria concentration significantly decreases. At the third sampling time (20 pm), the temperature decreases, the humidity increases, and the human activities increase (Hameed et al., 2009). All of these lead to an increased concentration of bacteria.

The fungal concentration of the LPM in this study (6.57 × 10^2^–1.33 × 10^4^ CFU/m^3^) was significantly higher than that of the other regions, such as the concentration range of fungi in Lanzhou, China (3.15 × 10^2^–4.72 × 10^3^ CFU/m^3^) (Jun-You et al., 2009), and the concentration range of fungi in Yokohama, Japan (13–2.75 × 10^3^ CFU/m^3^) (Togashi and Katsuki, 1952). Concentrations of fungi in the study did not fluctuate too much during the year (being higher in winter), which is not consistent with the results from previous studies. Most airborne fungi are thought to be derived from plants, but there are no plants in poultry markets (Fang et al., 2005). Thus, there is little influence on the concentration of airborne fungi, and the concentration does not vary a great deal. The diurnal variation of airborne fungi was similar to that of the bacteria, as mentioned above.

There were differences in the location of the microbial aerosol particles entering the respiratory system with different dynamic diameters. Aerosol particles with a diameter of 4.7–7 μm can enter the nasal cavity and upper airway; those of about 2.1–4.7 μm particle size can be deposited in small bronchial tubes, which leads to asthma and other diseases; and those with a diameter less than 2 μm can penetrate the alveolar into the respiratory tract and cause alveolar inflammation and other diseases (O’Connor et al., 2004). Grades five and six of the Anderson grade six sampler is equivalent to PM2.5 (Querol et al., 2004). After being inhaled into the human body it will go directly into the lungs, interfere with pulmonary gas exchange, and cause bronchitis, asthma, cardiovascular disease, and other diseases (Yao et al., 2002). In this study, the average distributions of bacteria, fungi, and Gram-negative bacteria in grade five and six of the Anderson sampler are 18.5, 19.5, and 14.0%, respectively. The percentage is relatively large, and it is a great threat to the health of people in LPM.

The contents of airborne endotoxin in the experiment was between 2.3 and 1.16 × 10^3^ EU/m^3^, not exceeding the standard of causing pneumonia (2000 EU/m^3^), but most of the content exceeded the level of having no impact on humans (100 EU/m^3^). Thus, long time exposure could affect the health of people in LPM. The level of endotoxin in the air was not correlated with the temperature (*P* > 0.05), but significantly correlated with relative humidity (*P* < 0.01, *r* = 0.9). Therefore, ventilation devices should be installed in LPM to reduce humidity, and thereby the content of endotoxin. Previous studies have demonstrated that some Gram-negative bacteria are relative to higher levels of endotoxin, such as Acinetobacter and *P. aeruginosa*, which were detected in this study. There was a weak correlation between them that is consistent with the previous experimental results (Hwang et al., 2011).

## Author Contributions

TC, YC, and LW designed experiments. BW and KM carried out experiments. BW and YC analyzed experimental results. BW, TC, and YC wrote the manuscript.

## Conflict of Interest Statement

The authors declare that the research was conducted in the absence of any commercial or financial relationships that could be construed as a potential conflict of interest.
